# Availability of appropriate airway monitoring at UK in-hospital cardiac arrest

**DOI:** 10.1186/cc14283

**Published:** 2015-03-16

**Authors:** S Turle, PB Sherren, T Callaghan, S Nicholson, SJ Shepherd

**Affiliations:** 1 Pan-Thames Intensive Care Training Scheme, London, UK; 2 KSS Intensive Care Training Scheme, London, UK

## Introduction

Airway complications are more common outside the operating theatre and in emergency situations. Capnography remains the gold standard of confirming correct endotracheal tube (ETT) placement, retaining high sensitivity and specificity in cardiac arrest [1]. The 2010 European Resuscitation Council guidelines for adult advanced life support recommended waveform capnography in this setting [2]. Failure to use capnography was also identified as a major contributor to airway-related morbidity and mortality in a national UK audit [3]. We sought to investigate current practice relating to the availability and use of capnography equipment cardiac arrest within UK hospitals.

## Methods

Between June and November 2014, a telephone survey was conducted of all UK acute hospitals with adult level 3 ICUs and an emergency department (ED). Hospitals were identified using nationally available data. A standardised telephone questionnaire was developed examining practice regarding intubation for cardiac arrest and the availability and utilisation of capnography within the ED, ICU and general wards. Questions were directed at the anaesthetist or intensive care doctor 'responding to cardiac arrest calls'. The respondent was given the option to decline participation. All data were anonymised.

## Results

A total of 211 hospitals met the inclusion criteria. The response rate was 100%. Arrest calls were mainly attended by anaesthesia (47.8%) and ICU doctors (38.3%) with around 2% physicians only. Most were a registrar grade (56.3%). The ability to measure ETCO_2_ was available in all but four EDs; most used waveform capnography. A similar pattern was seen was seen in the vast majority of ICUs: a single institution reported no capnography available. However, in 141 (66.8%) of the hospitals surveyed, no facility to measure ETCO_2_ was present on the general wards. Where available, 86.7% used capnography to confirm ETT placement. Less than 50% used ETCO_2_ to determine CPR effectiveness and 8% to prognosticate.

## Conclusion

We believe this is the first study of its kind to follow NAP4 and investigate the availability of capnography throughout for use during cardiac arrest. Whilst equipment levels appear adequate (albeit not perfect) in resuscitation areas, there appears a lack of availability of suitable devices on general wards.
